# 1298. High Prescription Rate of Medications with Significant Rifampin Drug Interactions in Patients with Diabetic Foot Osteomyelitis: Should Rifabutin be Included in Clinical Trials for Adjunctive Therapy?

**DOI:** 10.1093/ofid/ofad500.1137

**Published:** 2023-11-27

**Authors:** Christina Mallarino-Haeger, Allison Watson, Umnia Mahgoub, Lily Francis, Maryam Heydari, Muaaz Choudhary, Russell R Kempker, Marcos Schechter

**Affiliations:** Emory University School of Medicine, Atlanta, Georgia; Emory University, Atlanta, Georgia; Emory University, Atlanta, Georgia; Augusta University/University of Georgia Medical Partnership, Athens, Georgia; Emory University, Atlanta, Georgia; Lewis Katz School of Medicine, Temple University, Philadelphia, Pennsylvania; Emory University School of Medicine, Atlanta, Georgia; Emory University School of Medicine, Atlanta, Georgia

## Abstract

**Background:**

Diabetic foot osteomyelitis (DFO) is a leading cause of amputations. Rifamycins can penetrate osteoblasts and disrupt biofilm, making them ideal adjunctive antibiotics for DFO. A recent retrospective DFO study found that rifampin adjuvant therapy was associated with significantly higher amputation-free survival rate and randomized clinical trials to establish whether adjunctive rifampin therapy improves DFO outcomes are ongoing. Rifamycin use is complicated by drug-drug interactions (DDIs), primarily due to cytochrome P450 induction. However, rifabutin is a less potent and broad cytochrome P450 inducer versus rifampin. We evaluated DDI predicted rates of rifampin and rifabutin in a DFO cohort.

**Methods:**

We conducted a retrospective cohort study of all patients hospitalized with DFO between 2017 and 2019 at Grady Memorial Hospital (Atlanta, GA). We queried discharge billing records using ICD-10 codes for DFO and performed a chart review to confirm the diagnosis. We used the Lexicomp DDI tool to determine the number of potential DDIs between all medications prescribed upon discharge and rifampin or rifabutin.

**Results:**

There were a total of 530 hospital admissions among 330 unique patients for DFO of which 70% were male and 80% were Black. The mean hemoglobin A1c was 9.4%. Chronic kidney disease was present in 69% of patients. Among 239 DFO cases with a culture, *S. aureus* was identified in 79 (33%). Eighty-nine (27%) patients were prescribed medications with a class X (“avoid combination”) rifampin interaction versus 4 (1%) patients who were prescribed drugs with a rifabutin class X interaction. Two-hundred-and-forty (73%) patients had prescriptions for medications with a class D (“avoid combination”) rifampin interaction versus 15 (5%) with a class D rifabutin interaction. The most common medications with class X rifampin DDIs were proton pump inhibitors (20%) and directly acting oral anticoagulants (5.7%), while those for class D rifampin DDIs were atorvastatin (35%) and clopidogrel (12%).

**Conclusion:**

A high percentage of patients with DFO were prescribed medications with significant rifampin DDIs compared with rifabutin, supporting the need to investigate rifabutin for adjunctive DFO therapy.

**Disclosures:**

**All Authors**: No reported disclosures

